# Some pathological observations on the naturally infected dromedary camels (Camelus dromedarius) with the Middle East respiratory syndrome coronavirus (MERS-CoV) in Saudi Arabia 2018–2019

**DOI:** 10.1080/01652176.2020.1781350

**Published:** 2020-07-03

**Authors:** Abdelmohsen Alnaeem, Samy Kasem, Ibrahim Qasim, Ali Al-Doweriej, Ali Al-Houfufi, Abdulatif Alwazan, Abdalaziz Albadrani, Khuzayyim Alshaammari, Mohamed Refaat, Abdulkareem Al-Shabebi, Maged Gomaa Hemida

**Affiliations:** a Department of clinical studies, College of Veterinary Medicine, King Faisal University, Al-Hasa, Saudi Arabia; b Department of Veterinary Services, Ministry of Environment, Water and Agriculture, Riyadh, Saudi Arabia; c Department of Virology, Faculty of Veterinary Medicine, Kafrelsheikh University, Kafrelsheikh, Egypt; d Department of Pathology, Animal Health Research Institute, Dokki, Cairo, Egypt; e Department of Pathology, Veterinary Diagnostic Laboratory, Ministry of Environment, Water and Agriculture, Al-Hasa, Saudi Arabia; f Department of Microbiology, College of Veterinary Medicine, King Faisal University, Al-Hasa, Saudi Arabia; g Department of Anatomy, College of Veterinary Medicine, King Faisal University, Al-Hasa, Saudi Arabia

**Keywords:** Dromedary camel, Camelus dromedarius, MERS-CoV, natural infection, pathology, immunohistochemistry, prevalence

## Abstract

**Background:**

The natural MERS-CoV infection in dromedary camels is understudied. Recent experimental studies showed no obvious clinical signs in the infected dromedary camels.

**Aim:**

To study the pathological changes associated with natural MERS-CoV infection in dromedary camels.

**Methods:**

Tissues from three MERS-CoV positive animals as well as two negative animals were collected and examined for the presence of pathological changes. The screening of the animals was carried out first by the rapid agglutination test and then confirmed by the RT-PCR. The selected animals ranged from six to twelve months in age. The sensitivity of the latter technique was much higher in the detection of MERS-CoV than the Rapid test (14 out of 75 animals positive or 18% versus 31 out of 75 positive or 41%).

**Results:**

MERS-CoV induced marked desquamation of the respiratory epithelium accompanied by lamina propria and submucosal mononuclear cells infiltration, epithelial hyperplasia in the respiratory tract, and interstitial pneumonia. Ciliary cell loss was seen in the trachea and turbinate. In addition, degeneration of glomerular capillaries with the complete destruction of glomerular tufts that were replaced with fibrinous exudate in renal corpuscles in the renal cortex were noticed. Expression of the MERS-CoV-S1 and MERS-CoV-N proteins was revealed in respiratory tract, and kidneys.

**Conclusion:**

To our knowledge, this is the first study describing the pathological changes of MERS-CoV infection in dromedary camels under natural conditions. In contrast to experimental infection in case of spontaneous infection interstitial pneumonea is evident at least in some affected animals.

## 1. Introduction

The MERS-CoV emerged on the Arabian Peninsula in late 2012 (Zaki et al. 2012). According to the last WHO reports, there are 2494 laboratory-confirmed cases in humans in 27 countries across the world. About 858 (34%) of these patients had passed away as per November 2019 (World Health Organization (December 5, 2019). Dromedary camels remained the only known reservoir for MERS-CoV (Hemida et al. 2017b). The virus was isolated from many dromedary camel herds on the Arabian Peninsula and Africa (Chan et al. 2014; Hemida et al. 2017a; Ommeh et al. 2018; Younan et al. 2016; Yusof et al. 2017; Zaki et al. 2012). Dromedary camels remain the well-known reservoir of this virus. Other members of the family *Camelidae* such as alpaca and Illama and recently Bactrain camels were found to be susceptible for infection with the HCoV-EMC/2012 strain of the MERS-CoV (Adney et al. 2016; Adney et al. 2019; Adney et al. 2014; Crameri et al. 2016; Haverkamp et al. 2018; Khalafalla et al. 2015). MERS-CoVs from dromedary camels were proved to have the full potential to infect the human airway epithelium both *in vitro* and *in ex-vivo* models (Chan et al. 2014). There are many pieces of evidence about the possibility of the transmission of MERS-CoV between dromedary camels and humans (Azhar et al. 2014a, 2014b; Kasem et al. 2018). Several studies were conducted to document the course of MERS-CoV infection in dromedary camels, alpaca and llamas as the closely related members of the family *Camelidae* (Adney et al. 2016; Adney et al. 2014; Crameri et al. 2016; Haverkamp et al. 2018; Te et al. 2019). Some other studies reported the presence of mucopurulent discharges from the nose and lacrimation of the infected animals (Khalafalla et al. 2015). These results are consistent with the experimental MERS-CoV infection in dromedary camels. The infected animals showed a mild elevation in the body temperature at two days post-infection (dpi) as well as 5–6 days dpi (Adney et al. 2016; Adney et al. 2014). Meanwhile, the only reported visible clinical signs were mild rhinorrhea in all infected animals (Adney et al. 2016; Adney et al. 2014). The same research group reported the detection of virus particles in the nasal passage of the infected animals for seven dpi while the viral RNAs were still detectable up to 35 dpi (Adney et al. 2016; Adney et al. 2014).

Recent research reported some loss of the respiratory epithelium plus depletion of the viral receptors in the respiratory tract of the experimentally infected camels with MERS-CoV after the vaccination with the recombinant MERS-CoV/Vaccinia-Virus Ankara (Haverkamp et al. 2018). Although the natural MERS-CoV infection and pathogenesis in man were studied (Alsaad et al. 2018; Baseler et al. 2016b; van den Brand et al. 2015; Zhou et al. 2015), little information is known about the natural infection of MERS-CoV in dromedary camels yet. One abattoir surveillance was conducted in the eastern province of Saudi Arabia. This study revealed high prevalence of MERS-CoV nucleic acids in tissues collected from dromedary camels particular lung (63%) (Khalafalla et al. 2015). However, this study did not consider any pathological work on the positive tissues.

The main goals of the current study were to identify several naturally MERS-CoV infected dromedary camels, to study the pathological changes induced by the virus in these animals and to study the viral localization in various organs particular the respiratory tract (nasal turbinate, trachea, and lung) as well as kidneys. To the best of our knowledge, this study is among the first to document the pathological changes of naturally infected camels with MERS-CoV under field conditions. Further studies are needed for a better understanding of the molecular pathogenesis of MERS-CoV in its natural reservoir, the dromedary camel.

## 2. Materials and methods

### 2.1. Ethical statement and slaughtering procedure

We conducted this study according to the guidelines of the Animal Ethics protocols and the National Committee of Bio-Ethics, King Abdul-Aziz City of Science and Technology, Royal Decree No. M/59 (http://www.kfsh.med.sa/KFSH_WebSite/usersuploadedfiles%5CNCBE%20Regulations%20ENGLISH.pdf). Meanwhile, the sampling protocol was approved by the Ethics Committee of the Ministry of Environment.

### 2.2. The study design and sampling protocol

This study was conducted as a part of molecular surveillance of MERS-CoV among dromedary camels from March to April 2018 at the Ministry of Environment, Water, and Agriculture (MEWA), Riyadh, in collaboration with the King Faisal University, Saudi Arabia. A total of 75 dromedary camels were examined in the south Riyadh slaughterhouse (Supplementary Table 1). These animals were submitted for the regular slaughtering procedures for meat production. These animals were brought from the local markets close to the same slaughter house mainly by walking. Other animals were just transported for a very short time from their orgin to this slaughterhouse. The admitted animals were quarantined in a separate area in the slaughterhouse. During this time, these animals were carefully observed by veterinarians for the development of any obvious clinical signs if any. We marked these animals with a permanent marker to identify them in addition to their ear tag identification. These animals were subjected to the routine antemortem inspection in the slaughterhouse. The target animal population was less than two years old. Two nasal swabs were collected per each animal; the first was collected on the buffer of rapid MERS-CoV Ag detection kit while the second was collected on the viral transport medium (COPAN Italia, Brescia, Italy), and tested by the real-time PCR technique (RT-PCR). All collected swabs were transferred to the Riyadh veterinary laboratory within 2 hours after collection to confirm the presence of the MERS-CoV-RNA by RT-PCR.

**Table 1.** Lesion scoring of the MERS-CoV natural infected dromedary camels.

Organ	Lesion	Animal no		
		1	2	3
Nasal turbinate	Exfoliation of the epithelial cells (Fig. 2A)	+++	+	++
	Propria-submucosal lesions (Figs. 2B and C)	+++	+	++
	Focal areas of epithelial hyperplasia	+	+	+
Trachea	Partial loss of cilia, epithelial vacuolation and intra-epithelial infiltration with neutrophils (Fig 2D)	–	–	++
Bronchi	Hyperplasia of bronchial epithelia	–	+	+
Lungs	Interstitial pneumonia (Figs. 2E and Fig 3)	++	+++	+++
	Bronchopneumonia with many segmented neutrophils in bronchioli and alveoli	–	+++	–
Spleen	chronic hyperplasia with widened red pulp and depleted white pulp	–	–	+
Kidney	Glomerular and tubular lesions (Fig 2F).	++	+	++

Three positive MERS-CoV dromedary camels having very strong bands in the agglutination test (Supplementary Figure 1) as well as having the lowest Ct values among tested animals (16, 17, and 16) were selected for the downstream pathological investigations. These three animals were two males aged 6 and 12 months and an 8-month-old female. Meanwhile, two other negative MERS-CoV animals, were selected for further examination. These two control animals were a 6-month-old female and a 12-month-old male. The selected animals were slaughtered as previously described (Al-Owaimer et al. 2014).

**Figure 1.**
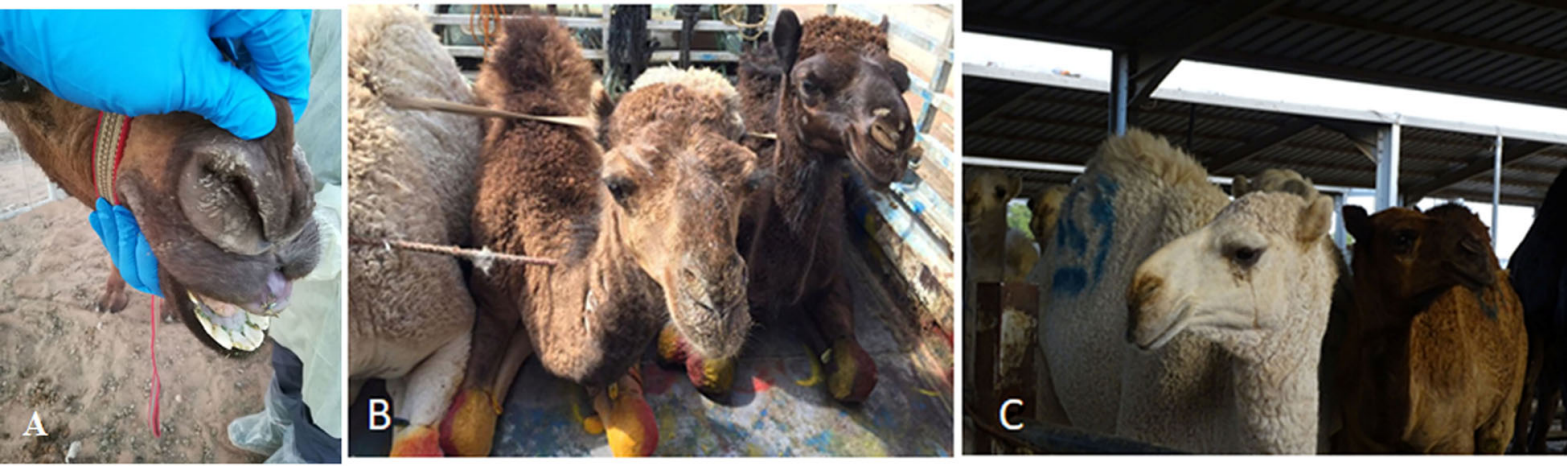
Signs of the naturally infected dromedary camels. Examination of some dromedary camels before slaughtering in the south Riyadh slaughterhouse and the protocol for the collection of nasal swabs **(A)**. Dromedary camel animals are showing mild nasal discharges and rhinorrhea **(B)**, dromedary camel showing unilateral lachrymation **(C)**.

### 2.3. Detection of MERS-CoV antigen in the nasal swabs by the rapid MERS-CoV antigen detection kit

The BIONOTE® Rapid MERS-CoV (BioNote Inc, Hwaseong, Gyeonggi, Republic of Korea) kit was used to test camel swabs. This technique was performed according to the manufacturing protocol. The test was considered negative when only the control (C) line appeared, whereas it was deemed to be positive when both the test line (T) and the control line (C) appeared. In the absence of the control line (C), the test was considered invalid.

### 2.4. Detection of MERS-CoV nucleic acids in the nasal swabs by RT-PCR

MERS-CoV RNAs were extracted from nasal samples by Qiagen viral RNA extraction kit, according to the manufacturer’s protocol (Qiagen GmbH, Hilden, Germany). The RT-PCR targeting using the upstream primers (Up-E) of MERS-CoV was used for initial screening (Hemida et al. 2020). Confirmation was done using the open reading frame (ORF) 1a. Five µl of the extracted RNA was subjected to RT-PCR using UpE primers by means of a Light-Mix Molecular Dx MERS-CoV UpE kits (Roche, Roche Molecular Systems Inc, Berlin, Germany) according to the manufacturer’s protocol. All positive samples by the UpE assay were confirmed by the results of the ORF1a as previously described (Hemida et al. 2020).

### 2.5. Histopathological examination

The selected animals were subjected to necropsy examination after proved positive by real-time PCR. Tissues from the upper and the lower respiratory tract, retropharyngeal lymph node, bronchial lymph node, spleen, and kidney were fixed in 10% neutral-buffered formalin immediately after slaughtering for five days. Tissues were processed with a thickness of 4 μm, and paraffin sections were stained with Hematoxylin and Eosin (H&E) for histopathological examination as previously described (Bancroft and Cook 1994).

### 2.6. Immunohistochemistry

Tissue sections from the trachea, nasal turbinates, lungs, and kidneys were screened for the presence of the MERS-CoV-S1 and MERS-CoV-N by immunohistochemistry. We used the polyclonal rabbit anti-MERS-CoV-S protein, S1 (Sinobiological, Wayne, PA 19087, USA), and monoclonal mouse anti-MERS-CoV nucleocapsid (NC), (Sinobiological, catalog#40068-V08B, AFS88943.1, Met1-Asp413) to detect the MERS-CoV-S and MERS-CoV-N proteins, respectively. The technique was carried out as per the manufactures instructions (Technicon’s LTD, Tokyo, Japan).

## 3. Results

### 3.1. Surveillance of MERS-CoV among dromedary camels in Riyadh slaughterhouse 2018–2019

Our results showed that 14/75 or 18% of the tested animals were positive by the rapid agglutination test. However, 31/75 or 41% of the tested animals were positive by real-time PCR technique.

### 3.2. Inspection of dromedary camels under the MERS-CoV naturally infection

Inspection of some dromedary camels admitted for slaughtering at one abattoir in south Riyadh region revealed no characteristic pathognomonic signs. A very small number of animals showed some mild discharges from the nose as well as mild lachrymation (Figure 1). Furthermore, no characteristic respiratory signs were noticed.

### 3.3. Pathological changes in the MERS-CoV naturally infected dromedary camels

**Figure 2.**
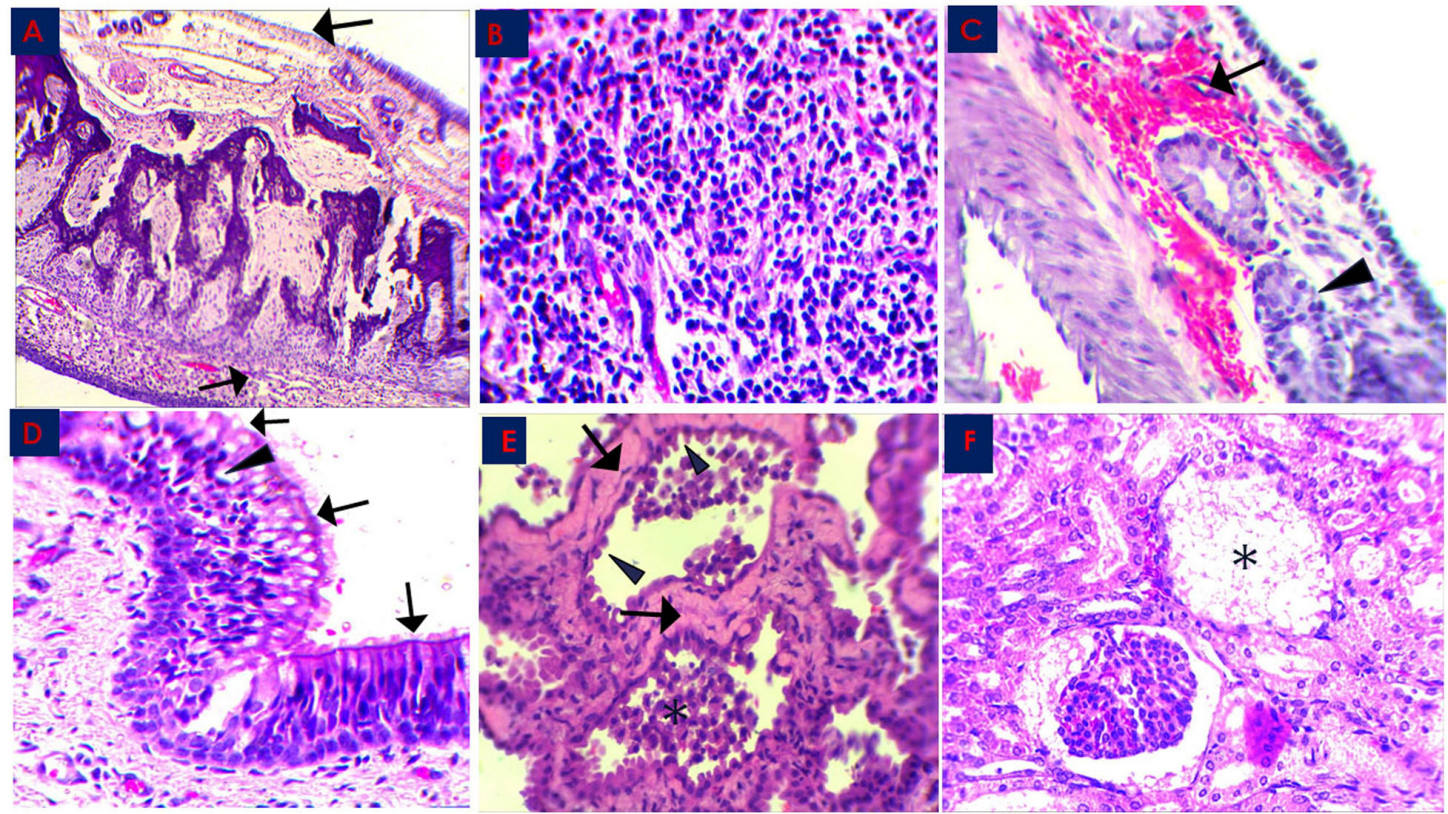
Histopathological changes in the MERS-CoV natural infected dromedary camels in Saudi Arabia 2018–2019. **A**: Nasal Turbinate showed exfoliation of the epithelial cells leaving denuded basement membrane (arrow), x100; **B**: Nasal Turbinate showed sub-epithelial mononuclear inflammatory cells infiltration (arrow), x400; **C**: Nasal Turbinate showed sub-epithelial hemorrhage (arrow) and glandular degeneration (arrowhead), x400; **D**: Trachea showed partial loss of cilia cells (arrow) and epithelial vacuolation (arrowhead), x400; **E**: Lung showed marked hyalinization of alveolar septa (arrow), with hypertrophy of pneumocytes type II (arrowhead) and intra-alveolar accumulation of macrophages (asterisk), x400; **F**: Renal Corpuscle showed marked fibrinous exudate with complete damage of glomerular tuft (asterisk), x400; all slides were stained with H&E stain.

**Figure 3.**
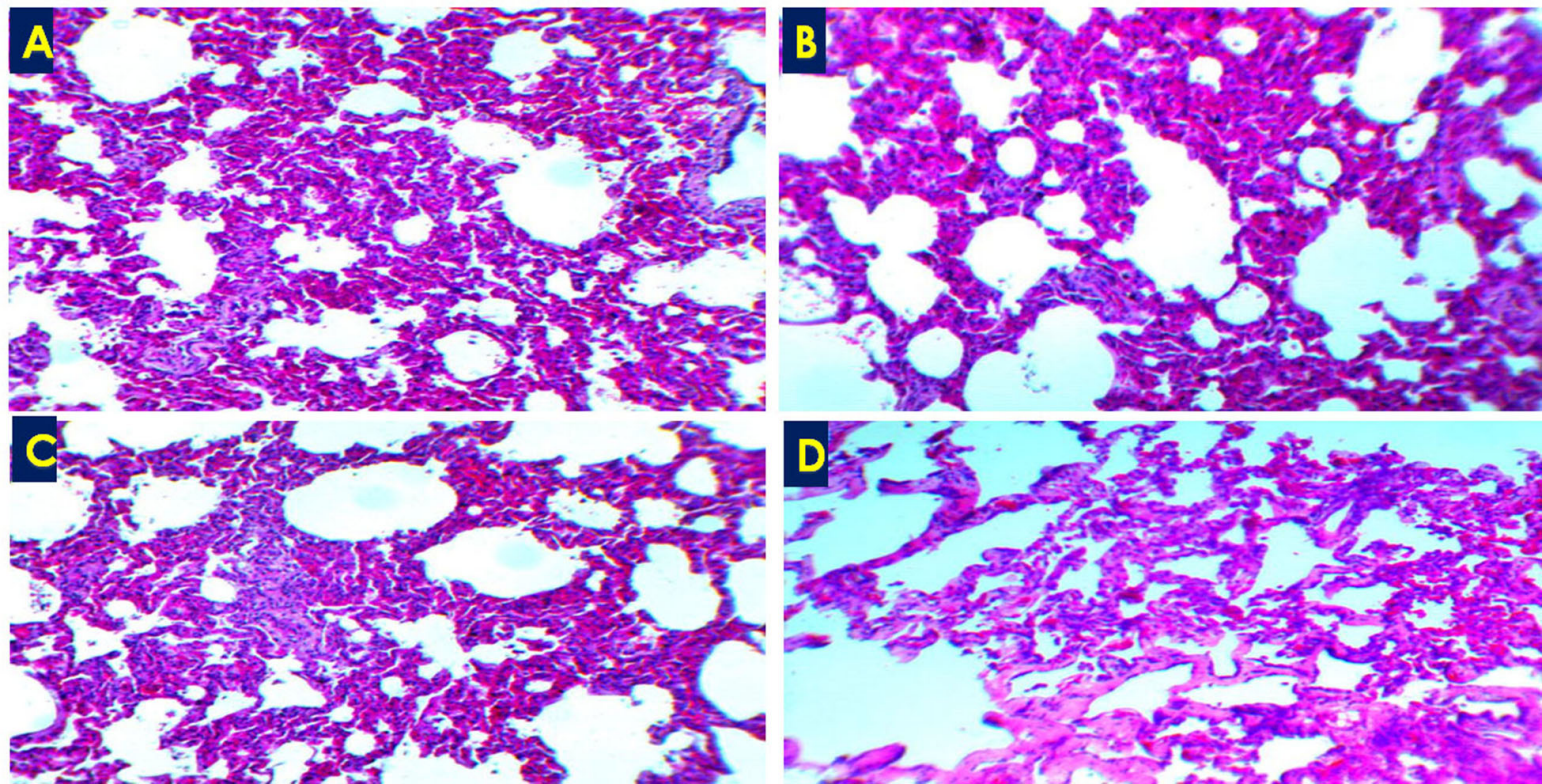
Acute interstitial pneumonia showed thickening of the alveolar wall due to leukocytic infiltration (A-C) and/or hyalinization (D), H&E ×100.

Microscopic examination of different tissues from the three RT-PCR-MERS-CoV positive animals showed variable lesions in the respiratory system and some (Figures 2 and 3 and Table 1). Most of the lesions of the respiratory organs were seen in the epithelial and propria-submucosal layers of nasal turbinate, trachea, and bronchi as well as interstitial tissues of the lungs. The nasal turbinate of the three animals showed exfoliation of the epithelial cells leaving denuded basement membrane with mononuclear cells’ infiltration (Figure 2(A,B)), hemorrhage, and glandular degeneration in the propria-submucosal layer (Figure 2(C)). Focal areas of mild hyperplasia were seen in the nasal turbinate epithelia of the three examined animals. The respiratory epithelium of the trachea in animal number three showed partial loss of cilia cells, epithelial vacuolation as well as a slight intra-epithelial infiltration with neutrophilic exocytosis (Figure 2(D)). Hyperplasia of bronchial epithelia was seen as well in animals’ number two and three. The lungs of the three animals revealed an acute interstitial pneumonia as many alveoli showed thickening of the alveolar septa with their infiltration with lymphocytes and other mononuclear inflammatory cells (Figure 3(A–C)). Other pulmonary alveoli showed marked proliferation of pneumocytes type II with alveolar septa hyalinization and presence of some macrophages in the alveolar lumen (Figures 2E and 3D). The spleen of animal number three showed chronic hyperplasia with widened red pulp and depleted white pulp. The kidney in the three animals showed degeneration of glomerular capillaries with the complete destruction of glomerular tufts that were replaced with a significant amount of fibrinous exudate in many renal corpuscles in one animal (Figure 2(F)). Many renal tubules showed degenerative changes. Focal interstitial hemorrhage was observed in renal parenchyma. Histopathological lesions’ score was summarized in Table 1.

### 3.4. MERS-CoV tissue localization and tropism in the naturally infected dromedary camels

**Figure 4.**
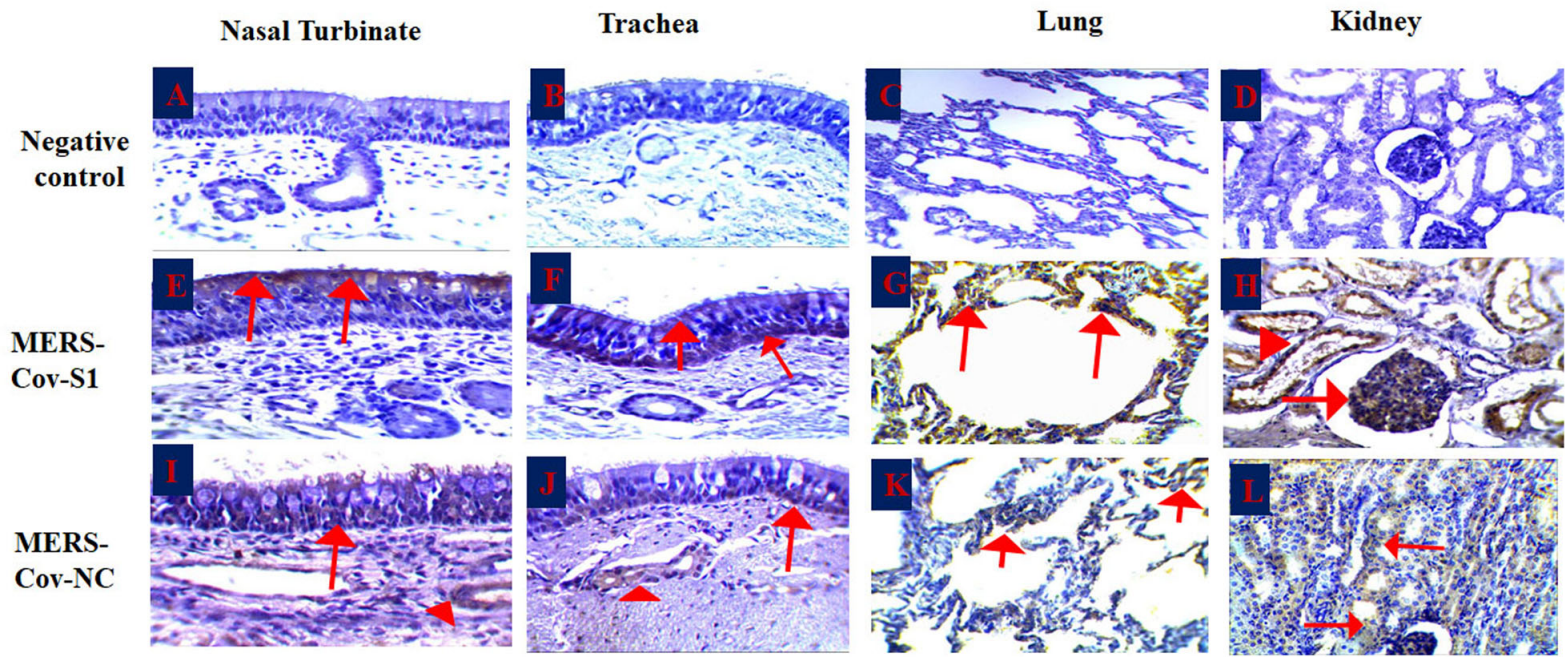
Immunohistochemistry of the MERS-CoV natural infected dromedary camels. A-D: Nasal turbinate, trachea, lungs and kidney respectively showed no signal; E-F: Nasal turbinate and trachea showed signals of MERS-CoV-S1 viral antigen in the epithelial surfaces; G: pulmonary alveoli showed signals of MERS-CoV-S1 viral antigen, H: MERS-CoV-S1 antigen was detected in both glomerular epithelia (arrow) and renal tubules (arrow head); I-J: Nasal turbinate and trachea showed weak signals of MERS-CoV-NC viral antigen in both epithelial surfaces (arrow) and sub-epithelial glands (arrowhead); K: Pulmonary alveoli showed weak signals of MERS-CoV-NC viral antigen; L: Renal tubules showed weak signals of MERS-CoV-NC viral antigen.

The IHC results of the examined tissues elucidated detectable signals of the MERS-Co viral antigens, spike 1 (S1), and nucleocapsid (NC) antigens (Table 2; Figure 4). The nucleocapsid (NC) viral antigen was detected in all examined tissues with weak signals (Figure 4(I–L)) when compared with the MERS-CoV-S1 antigen (Figure 4(E–H)). Signals of both viral antigens were compared with the negative control sections (4 A-4D). The evaluation of the signals depended on both the distribution as well as the strength of the reaction in the examined tissues. Most of the reactions were obviously detected in both the apical epithelial layers and submucosal glands of the nasal turbinate, trachea, and bronchi. The NC antigen reaction was detected in the basal epithelial cell layer and not in the apical epithelium of the trachea of one animal.

**Table 2.** Expression levels of the MERS-CoV-S1 and the MERS-CoV-N proteins in different organs of the naturally infected dromedary camels.

Organs	MERS-CoV-S1	MERS-CoV-NC
**Nasal turbinate**	**++**	**+**
**Trachea**	**++**	**+**
**Lungs & bronchi**	**++**	**+**
**Kidneys**	**++**	**+**

## 4. Discussion

The course of the natural MERS-CoV infection in dromedary camels is still not well studied yet. The main goals of the current study were to identify some naturally infected MERS-CoV dromedary camels to document the pathological changes in various body organs during the course of MERS-CoV infection. Epidemiological surveillance was conducted on 75 dromedary camels’ of various gender and age in one of the large abattoirs in the central Riyadh region. The majority of the animals under study were brought to the slaughterhouse from the local camel market which is closely to this abbatoir. This is the typical style of the camel markets and slaughter houses in Saudi Arabia (Hemida and Alnaeem 2019). Thus, these animals did not suffer from stress posed by the traveling as in the case of other species of large animals especially cattle (Van Engen and Coetzee 2018). The screening of the animals was carried out first by the rapid agglutination test and then confirmed by the RT-PCR. The sensitivity of the latter technique was much higher in the detection of MERS-CoV than the Rapid test (18% versus 41%).

Several studies reported the histological and immunohistochemical changes on some members of the family *Camelidae* (Adney et al. 2014; Baseler et al. 2016a; van den Brand et al. 2014; van Doremalen and Munster 2015). Experimental inoculation of dromedary camels with MERS-CoV has been shown to cause mild disease condition in infected animals. The pattern of this infection showed degeneration and necrosis with loss of pseudostratified respiratory epithelium in the upper and lower respiratory tract. This was in the absence of pneumonia. These studies also revealed positive viral antigens in respiratory tissues, particularly epithelium of nasal turbinate, which was considered as the primary site of virus replication (Adney et al. 2014). These findings are consistent with our findings in the upper respiratory tract (Haverkamp et al. 2018). We found mild to moderate segmental hyperplasia, partial loss of apical epithelial cells (erosion) of the nasal turbinate epithelium with lamina propria and submucosal infiltration of moderate numbers of lymphocytes and macrophages. This was in addition to intraepithelial exocytosis of neutrophilic granulocytes and ciliary cell loss of tracheal epithelium in MERS-CoV experimentally infected dromedaries.

Moreover, other studies detected rare foci of mucosal erosion of the nasal turbinate accompanied by minimal-to-mild sub-epithelial infiltration of neutrophils and macrophages and fewer lymphocytes in alpacas experimentally infected with MERS-CoV (Adney et al. 2016). In contrast to the absence of lesions among lungs in all the above studies, another study reported that histological lesions in MERS-CoV–infected macaques were limited to the lungs that showed thickening of alveolar septae by edema fluid, fibrin and type II pneumocytes hyperplasia (de Wit et al. 2013). They have demonstrated viral antigens in the lungs of all monkeys by the IHC technique as well as viral RNA through the *in situ* hybridization (de Wit et al. 2013).

The three examined animals in our study showed typical interstitial pneumonia, which was significantly similar to other studies in rhesus macaque (de Wit et al. 2013). Another study in rhesus macaques showed marked proliferation of the type II pneumocytes with hyalinization and thickening of the alveolar septae. This was in addition to infiltration with macrophages and neutrophils as well as the presence of some macrophages and fibrin in the alveolar lumen. However, we did not detect intra-alveolar fibrin threads in the three positive animals under study. The same study also detected viral antigens by an antibody specific for MERS-CoV within type I pneumocytes by the IHC technique (Prescott et al. 2018). The presence of the viral antigens in alveolar type I pneumocytes may explain their proliferation into type II pneumocytes and describe the consistent lesions of interstitial pneumonia. Fatal pneumonia has been reported in the common marmoset infected with MERS-CoV, which showed interstitial pneumonia and hemorrhages (Falzarano et al. 2014).

Our study confirms the mild clinical signs in the presence of some respiratory and kidney lesions in the three tested animals. This may explain and suggest the unique nature of resistance of dromedary camels to the natural MERS-CoV infection; thus, no visible or severe clinical signs displayed by the infected animals. One study reported the possibility of reinfection of the same animals and herds of camels with the MERS-CoV within a short period (Hemida et al. 2017a). This may contribute to the pathological lesions in the examined animals. Further large-scale field studies among pulmonary and renal involvement on the natural MERS-CoV tropism are required.

## Conclusions

The overall course of natural MERS-CoV infection is associated with mild clinical signs as well as lesions. However, further extensive studies are needed to explore the complete course of MERS-CoV infection, including detailed clinical observations, blood biochemistry as well as pathological changes involving other body systems.

## Supplementary Material

Supplemental Material

Supplemental Material
