# Supplementary material for: Some pathological observations on the naturally infected dromedary camels (Camelus dromedarius) with the Middle East respiratory syndrome coronavirus (MERS-CoV) in Saudi Arabia 2018–2019
Source: Vet Q. 2020 Jul 3;40(1):190–7. doi: 10.1080/01652176.2020.1781350 (PMC7734115; doi:10.1080/01652176.2020.1781350)
Supplement: Supplemental Material [file TVEQ_A_1781350_SM0720.docx]

**Supplementary table 1: Summary of age and sex of the tested animals**

| **N** | **Age** | **Sex** | **Target animals*** |
| --- | --- | --- | --- |
| 1 | 12m | Male |  |
| 2 | 8m | Male |  |
| 3 | 12m | Male |  |
| 4 | 14m | Male |  |
| 5 | 12m | Male |  |
| 6 | 16m | Male |  |
| 7 | 15m | Female |  |
| 8 | 12m | Female |  |
| 9 | 13m | Male |  |
| 10 | 12m | Female |  |
| 11 | 10m | Female |  |
| 12 | 10m | Male |  |
| 13 | 12m | Female |  |
| 14 | 6m | Male |  |
| 15 | 8m | Male |  |
| 16 | 8m | Male |  |
| 17 | 6 year | Female |  |
| 18 | 7 year | Female |  |
| 19 | 7 year | Female |  |
| 20 | 12 m | Male |  |
| 21 | 12m | Male |  |
| 22 | 14m | Male |  |
| 23 | 8m | Male |  |
| 24 | 8m | Male |  |
| 25 | 8m | Male |  |
| 26 | 10m | Male |  |
| 27 | 10m | Male |  |
| 28 | 12m | Male | PA (1) |
| 29 | 10m | Female |  |
| 30 | 12m | Male |  |
| 31 | 12m | Male |  |
| 32 | 13m | Male |  |
| 33 | 14m | Male |  |
| 34 | 12m | Female |  |
| 35 | 8m | Female | PA (2) |
| 36 | 8m | Female |  |
| 37 | 6m | Female |  |
| 38 | 6m | Male |  |
| 39 | 8m | Male |  |
| 40 | 6m | Male |  |
| 41 | 6m | Male | PA (3) |
| 42 | 8m | Male |  |
| 43 | 10m | Male |  |
| 44 | 12m | Male | NA (1) |
| 45 | 12m | Male |  |
| 46 | 12m | Male |  |
| 47 | 12m | Male |  |
| 48 | 10m | Male |  |
| 49 | 8m | Male |  |
| 50 | 8m | Male |  |
| 51 | 8m | Male |  |
| 52 | 7m | Male |  |
| 53 | 6m | Male |  |
| 54 | 8m | Male |  |
| 55 | 6m | Male |  |
| 56 | 7m | Male |  |
| 57 | 7m | Male |  |
| 58 | 10m | Male |  |
| 59 | 12m | Male |  |
| 60 | 12m | male |  |
| 61 | 8m | male |  |
| 62 | 10m | male |  |
| 63 | 10m | male |  |
| 64 | 8m | male |  |
| 65 | 6m | male |  |
| 66 | 8m | male |  |
| 67 | 8m | male |  |
| 68 | 12m | male |  |
| 69 | 12m | male |  |
| 70 | 6m | Female | NA (2) |
| 71 | 2y | male |  |
| 72 | 6m | male |  |
| 73 | 5m | male |  |
| 74 | 5m | male |  |
| 75 | 12m | Female |  |

* Animals selected for histopathological examination

PA=Positive animals

NA=Negative animals
