# Supplementary figures and images for: Some pathological observations on the naturally infected dromedary camels (Camelus dromedarius) with the Middle East respiratory syndrome coronavirus (MERS-CoV) in Saudi Arabia 2018–2019
Source: Vet Q. 2020 Jul 3;40(1):190–7. doi: 10.1080/01652176.2020.1781350 (PMC7734115; doi:10.1080/01652176.2020.1781350)

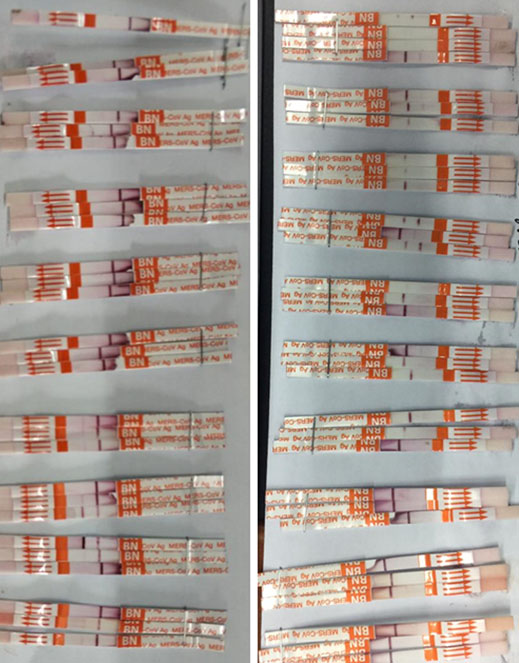

Supplement: Supplemental Material [file TVEQ_A_1781350_SM0702.jpg]
